# Optimization of Cockpit Ventilation for Polar Cruise Ships in Combination with Windscreen Defogging and Cabin Comfort Considerations

**DOI:** 10.3390/e24081061

**Published:** 2022-07-31

**Authors:** Hong Shi, Qianwei Zhang, Wenbing Xu, Meinan Liu, Jiashuang Pan, Jie Yuan, Kaijie Yang

**Affiliations:** 1 College of Energy & Power Engineering, Jiangsu University of Science and Technology, 2 Mengxi, Jingkou, Zhenjiang 212003, China; shihong@nuaa.edu.cn (H.S.); zqw1054200681@163.com (Q.Z.); liumeinan98@foxmail.com (M.L.); jackpanchinese@foxmail.com (J.P.); 2 Key Laboratory of Aircraft Environment Control and Life Support, MIIT, Nanjing University of Aeronautics & Astronautics, 29 Yudao Street, Nanjing 210016, China; jieyuannuaa@foxmail.com (J.Y.); tiehanhan@nuaa.edu.cn (K.Y.)

**Keywords:** cockpit, polar cruise ship, entropy weight–TOPSIS, defogging, comfort

## Abstract

Polar cruise ships are exposed to extreme external conditions during voyages, resulting in cockpit windscreens that are prone to fogging and frosting, seriously affecting the driver’s vision and even threatening navigation safety. However, the current research mainly focuses on cabin comfort, ignoring the coupling of defogging and comfort. Accordingly, this paper combines cockpit-windshield-defogging design and cockpit comfort considerations, and proposes 108 orthogonal-ventilation design parameters based on the four basic ventilation methods. The effects of different air supply parameters on comfort and anti-fog characteristics are investigated by using fluid dynamics simulation methods. The entropy weight–TOPSIS algorithm is employed to find the optimal ventilation parameters. The results show that the “Down-supply up-return type vertical jet” air supply method corresponding to an air supply velocity of 1 m/s, an air supply temperature of 297 K, and an air supply relative humidity of 30% has the smallest Euclidean distance $d_{i}^{+}$ from the positive ideal solution, and the largest Euclidean distance $d_{i}^{-}$ from the negative ideal solution; thus, it obtains a higher $c_{i}$ and the highest priority. This air supply method provides the best thermal comfort for the drivers, as well as the best anti-fogging and defogging effect. The results can be useful to provide suggestions for the future design of the air-conditioning systems in polar cruise ships.

## 1. Introduction

Polar cruises have become a major means of transport for people travelling to the polar regions for tourism, research, and expeditions. In general, polar cruise ships have higher requirements for safety, comfort, and environmental friendliness when crossing rough seas than regular cruise ships [1]. However, the harsh weather in the polar regions make the cockpit windscreen susceptible to fog and frost, which can seriously impair the driver’s visibility and, thus, compromise safety during the cruise. Additionally, in polar cruise ships, the driver’s physical health and work efficiency depend to a great extent on the comfort of the cabin [2,3]. Therefore, a good air ventilation system not only addresses functional needs such as cabin defogging, but also creates a thermally comfortable micro-environment for the drivers [4].

Some research has been carried out in the area of windscreen fogging and cabin comfort performance studies. In the studies of windscreen-defogging characteristics, Leriche et al. [5] developed a droplet distribution model that predicts atomization patterns and investigated the effects of relative humidity, air temperature, wall temperature and surface condition on temperature changes. It was found that the relative humidity is the most important factor for fogging and defogging. Aroussi et al. [6] used FLUENT to simulate the velocity and temperature field of the windscreen and the effect of a defrost jet on passenger comfort. It was found that the defrost jet and the obstacle inside the cabin jointly determined the characteristics of the flow field. However, these two factors did not have a significant impact on passenger comfort. Willenborg et al. [7] studied the flow characteristics of the airflow in a defogging device and used a hot-wire anemometer to measure the wind velocity near the nozzle and on the inner surface of the windscreen. Zhang [8] proposed the “rain falling” air-conditioning system, and pointed out that the cabin air-conditioning system and glass-defogging system should be a combined design. You et al. [9] simulated the moisture condensation of a chamber model to predict its inner hydrothermal distribution in a high-humidity climate. Yang et al. [10] studied the effect of defogging airflow velocity on liquid film thickness and found that when the defogging airflow velocity was greater than 0.6 m/s, the dew on the front window decreased rapidly.

In addition, thermal comfort has become one of the most important criteria for evaluating the ship environment. Most comfort studies of cabin air-conditioning systems are based on thermal-comfort indicators such as PMV, and Ahola and Mugge [11] found that the human body can withstand higher wind velocities at higher room temperatures, and suggested that it is better adapted to lower wind velocities at lower room temperatures. Somnath and Mayur [12] analyzed the thermal comfort and airflow distribution inside a vehicle cabin. It was found that the modified grille allows for a more uniform flow field distribution and better cooling. It is also shown that the flow field strongly affected the overall cooling rate. Danca et al. [13] suggests that relatively humidity between 30% and 70% does not have an influence on the thermal comfort at neutral temperatures. Zhang et al. [14] carried out numerical simulations of the internal environment of a car model. It was found that in order to achieve thermal comfort, the cabin temperature should be between 297 K and 302 K and the local velocity should not be greater than 0.5 m/s. Xia et al. [15] used two evaluation indicators, the PMV-PPD value and the average air age, to study the effect of radiant-cooling terminal arrangement on the thermal environment of cruise ship cabins.

The above studies show that scholars have achieved some research results in the area of cockpit defogging and comfort in cabins [16,17]. However, there is relatively little research literature on coupling defogging and comfort performance at the same time. As the cockpit windscreen is relatively close to the human driving position, its air supply characteristics have a direct impact on the comfort of the driver. Therefore, it is urgent to explore an optimal ventilation system to achieve the balance between cruise anti-fog and comfort. 

In order to ensure the safe navigation of polar cruise ships and to enhance the comfort of the driver, the anti-fog effect of the cockpit windscreen was investigated by using the commercial CFD program FLUENT. In this paper, four basic ventilation methods and different air supply parameters (air supply velocity, air supply temperature and air supply relative humidity) were studied. Additionally, 108 groups of working conditions were obtained for the orthogonal combinations of the parameters. In addition, the optimal air supply parameters are obtained based on the entropy weight–TOPSIS method. The results of this paper provide a technical reference for the study of anti-fog characteristics and personnel comfort of cruise ships.

## 2. Geometric Model and Methodology

### 2.1. Geometric Parameters of the Cockpit

**Figure 1:**
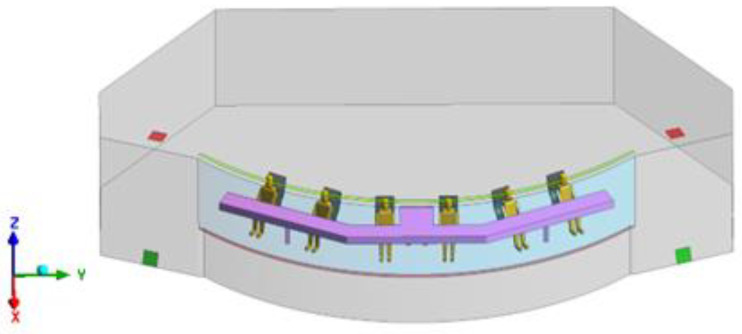
Model of a polar cruise ship.

The geometric model of the polar cruise ship is given in Figure 1 and the dimensional parameters are shown in Table 1.

**Figure 2:**
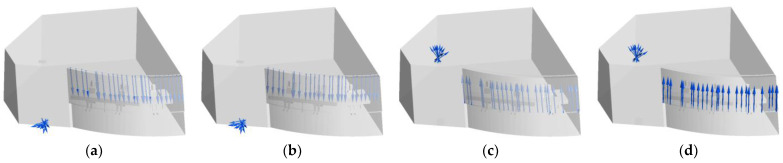
Ventilation methods. (**a**) Top-supply down-return type window-attached-jet (TSDR-WAJ); (**b**) Top-supply down-return type vertical jet (TSDR-VJ); (**c**) Down-supply up-return type window-attached-jet (DSUR-WAJ); (**d**) Down-supply up-return type vertical jet (DSUR-VJ).

Four ventilation methods are proposed in this paper, as shown in Figure 2, and the setting of boundary conditions is shown in Table 2.

### 2.2. Algorithm Validation

**Figure 3:**
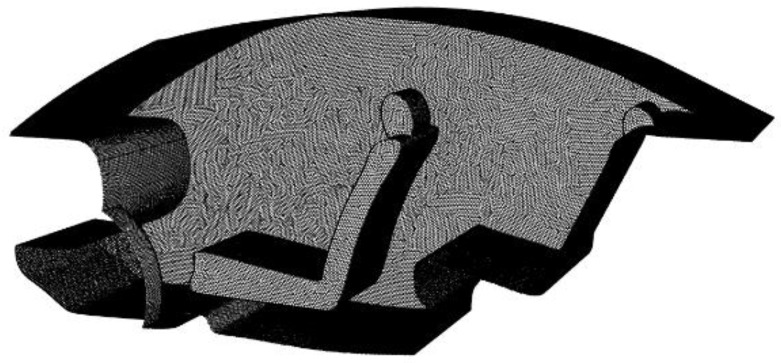
Models and cells.

**Figure 4:**
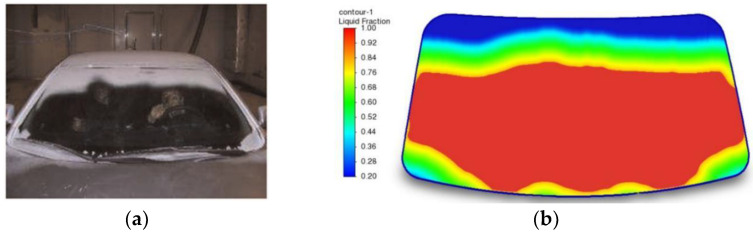
Comparison of experimental and simulated conditions. (**a**) The experimental results; (**b**) The numerical results.

To ensure the reliability of the simulation method in this paper, the experimental results of defrosting in the literature [18,19] were used for comparison with the numerical simulation results, where the model dimensions of the computational domain are 3.1 m × 1.8 m × 1.2 m (length × width × height), and the windscreen thickness is 4.96 mm, as shown in Figure 3. Assume uniform frost on the outside surface of the windscreen, an ambient temperature of −18 °C, and a frost layer thickness of 0.5 mm. A comparison of the experimental and simulated results of defrosting at 20 min is shown in Figure 4.

As shown in Figure 4, the simulation results are in good agreement with the experimental results. However, the defrosting effect is not exactly the same because there is some discrepancy between the computational-domain dimensions and the defrost inlet dimensions. In general, the simulation results do not differ significantly from the experimental results. Therefore, the simulation method for defrosting and defogging in this paper has a certain reliability and can be used for subsequent studies.

### 2.3. Mesh Independence

It is worth noting that the left and right parts of the cockpit model are completely symmetrical in this paper. In order to save computational resources, the left part of the model is meshed and computed in this paper. After the calculation is completed, the results of the whole model are then displayed in a symmetric approach. 

**Figure 5:**
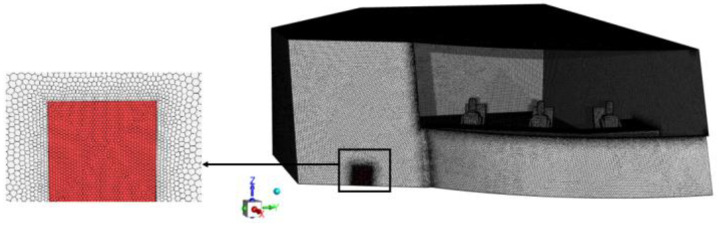
Mesh of the cockpit geometry model.

In this section, unstructured polyhedral meshes are generated for the cockpit model and the meshes are refined for places with intense flow changes. To ensure that the results of this paper are independent of the number of cells, mesh independence verification is carried out before the numerical calculations. The mesh of the cockpit geometry model and the locally enlarged mesh are shown in Figure 5.

**Figure 6:**
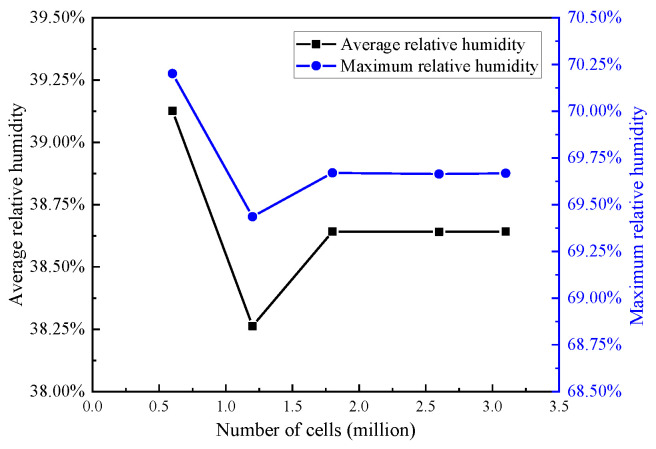
Results of mesh independence verification.

As an example, the air supply method is “Down-supply up-return type vertical jet”, the air supply velocity is 1 m/s, the air supply temperature is 297 K and the air supply relative humidity is 30%. The average relative humidity and the maximum relative humidity of the windscreen surface were used as evaluation indicators, and the results are shown in Figure 6.

As shown in Figure 6, when the number of cells was larger than 1.8 million, the average relative humidity and the maximum relative humidity of the windscreen surface tended to be stable and did not change significantly with the number of cells. Therefore, a mesh of 1.8 million was employed for the numerical simulation in this paper, while ensuring the accuracy of the mesh and considering the computational cost.

## 3. Performance Evaluation Indicators

### 3.1. Comfort Evaluation Indicators

1. PMV

The PMV model was proposed by Fanger [20] based on the ASHRAE 7-point scale and corresponds to the thermal-comfort indicator as shown in Table 3. 

The human-body-heat-balance equation is calculated as follows.

(1) $$\mathrm{PMV}=\left[0.303\cdot \exp\left(-0.036\cdot M\right)+0.028\right]\cdot L$$

(2) $$\begin{array}{l} L=M-W-3.96\cdot 10^{-8}\cdot f_{cl}\cdot \left[{\left(T_{cl}+273\right)}^{4}-{\left(T_{r}+273\right)}^{4}\right]\\ -f_{cl}\cdot h_{c}\cdot \left(T_{cl}-T_{a}\right)-3.05\cdot 10^{-3}\cdot \left[5733-6.96\cdot \left(M-W\right)-P_{a}\right]\\ -0.42\cdot \left(M-W-58.15\right)-1.7\cdot 10^{-5}\cdot M\cdot \left(5876-P_{a}\right)\\ -0.0014\cdot M\cdot \left(34-T_{a}\right) \end{array}$$

where *M* is the metabolic volume, *W* is the external work output of the passenger, *f_cl_* is the ratio of the surface area of the garments to the surface area of the naked body, *T_cl_* is the external surface temperature of the garments, *T_r_* is the average radiant temperature, *h_c_* is the surface heat transfer coefficient between the garments and the air, *T_a_* is the air temperature, *P_a_* is the partial pressure of water vapor.

2. DR

A blowing sensation is the most common factor causing local discomfort, which depends on air temperature, velocity and turbulence intensity. Fanger et al. [21] developed a model of the blowing dissatisfaction rate DR as a function of air temperature, velocity and turbulence intensity, which is predicted as follows.

(3) $$DR=\left[\left(34-T_{a}\right)\cdot {\left(u-0.05\right)}^{0.62}\cdot \left(0.37\cdot u\cdot Tu+3.14\right)\right]$$

where *T_a_* is the temperature of air, *u* is the average air velocity, and *Tu* is the turbulence intensity of air.

**Figure 7:**
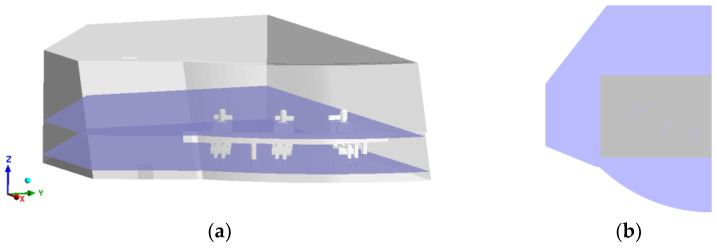
Thermal-comfort evaluation area. (**a**) Surface of Z = 1.0 m and Z = 0.2 m; (**b**) Selected area.

According to the ASHRAE standard, the blowing sensation should be less than 20% and the lower the blowing sensation, the better the thermal comfort. Considering that the cockpit of a polar cruise ship is a large space, it is difficult to ensure its global comfort and the driver’s position in the cockpit is relatively fixed, so this paper proposes a local thermal-comfort evaluation method to ensure the thermal comfort of the driver. Figure 7 shows the thermal-comfort evaluation area proposed in this paper.

As shown in Figure 7a, two typical surfaces are selected in the Z-direction, i.e., Z = 1.0 m (driver’s shoulder) and Z = 0.2 m (driver’s foot). As shown in Figure 7b, the grey areas are the selected evaluation areas.

In summary, there are four evaluation indicators for thermal comfort in the cockpit of a polar cruise ship: the mean PMV and mean DR values for the area near the driver on the Z = 1.0 m surface and the mean PMV and mean DR values for the area near the driver on the Z = 0.2 m surface.

### 3.2. Anti-Fog Evaluation Indicator

Considering that fogging will occur when the relative humidity of the air reaches 100%, in order to allow for a certain design margin, 95% was chosen as the upper limit in this paper, which means that fogging is considered to occur when the relative humidity of the air is above 95% in the steady-state calculation. At the same time, this paper stipulates that when the fogging area of the windscreen is less than 5% of the total area, then the basic requirement of anti-fog is satisfied. Based on meeting the basic requirements, the lower the average value of relative humidity on the inside of the windscreen, the better the anti-fog effect.

### 3.3. Fog Elimination Evaluation Indicator

In this paper, the model of Eulerian wall film in FLUENT software is used for the defogging calculation. In the defogging calculation process, the fog layer has a certain thickness at the initial moment, and when the ventilation system is activated, the fog layer thickness is below 1 × 10^−20^ m, and the defogging is completed. In addition, this paper considers that the defogging operation is completed when the defogging area is greater than 95% of the total windscreen area. Therefore, the shorter the defogging completion time, the better the defogging effect.

## 4. Effect of Different Air Supply Methods on Comfort and Anti-Fogging Characteristics

**Figure 8:**
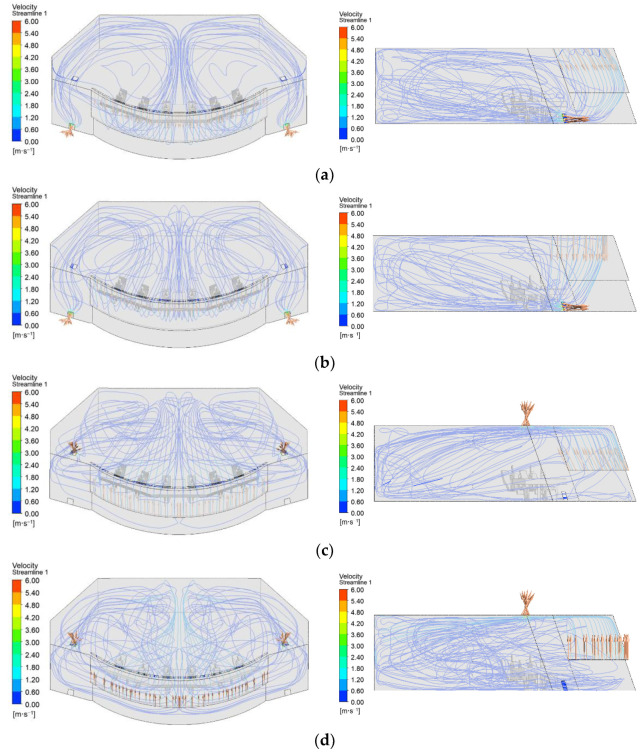
Flow field distribution under different air supply methods. (**a**) Top-supply down-return type window-attached-jet (TSDR-WAJ); (**b**) Top-supply down-return type vertical jet (TSDR-VJ); (**c**) Down-supply up-return type window-attached-jet (DSUR-WAJ); (**d**) Down-supply up-return type vertical jet (DSUR-VJ).

In this section, the influence of different air supply methods on the comfort and anti-fog characteristics is analyzed in detail, using the air supply velocity of 1 m/s, air supply temperature of 297 K and air supply relative humidity of 30% as benchmarks. Figure 8 shows the distribution of the flow field corresponding to the different air supply methods.

As shown in Figure 8a,b, the flow field of the “Top-supply down-return type window-attached-jet” air supply method is approximately the same as that of the “Top-supply down-return type vertical jet” air supply method. Obviously, the airflow first moves down the windscreen and then gradually towards the rear of the cockpit, forming vortices at the dead ends on the left and right sides and eventually exiting through the lower exhaust air outlet. The difference is that the airflow is more closely aligned with the windscreen in the “Top-supply down-return type window-attached-jet” ventilation system than in the “Top-supply down-return type vertical jet” ventilation system.

As shown in Figure 8c,d, the flow field of the “Down-supply up-return type window-attached-jet” and “Down-supply up-return type vertical jet” air supply methods differ significantly from the flow fields of the previous two air supply methods, and the streamlines are quite chaotic.

**Figure 9:**
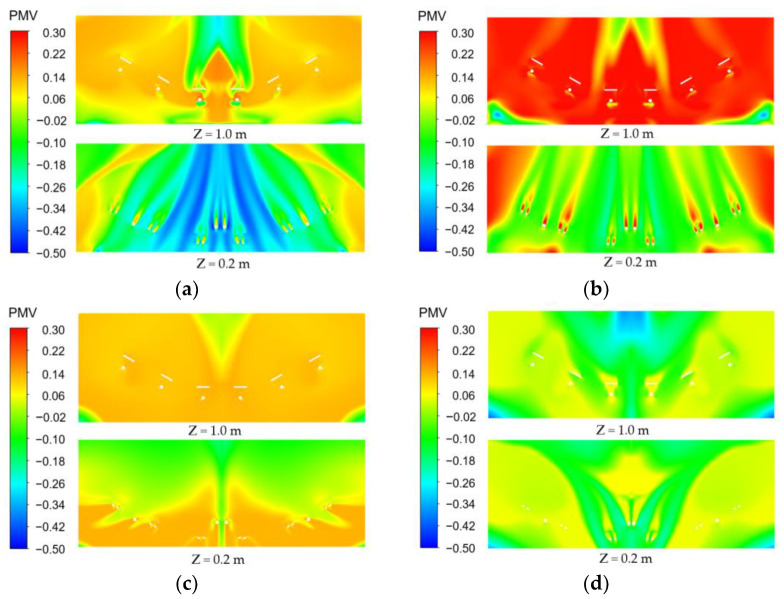
PMV value distribution under different air supply methods. (**a**) Top-supply down-return type window-attached-jet (TSDR-WAJ); (**b**) Top-supply down-return type vertical jet (TSDR-VJ); (**c**) Down-supply up-return type window-attached-jet (DSUR-WAJ); (**d**) Down-supply up-return type vertical jet (DSUR-VJ).

**Figure 10:**
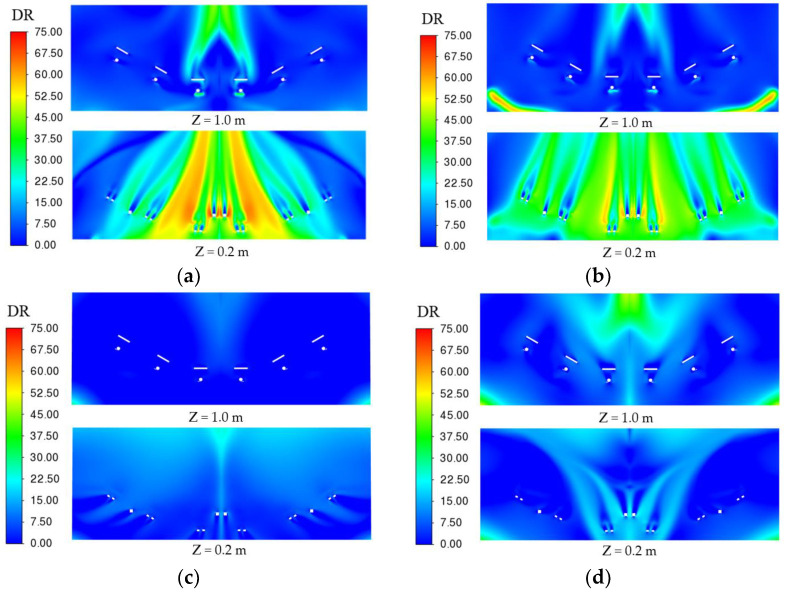
DR value distribution under different air supply methods. (**a**) Top-supply down-return type window-attached-jet (TSDR-WAJ); (**b**) Top-supply down-return type vertical jet (TSDR-VJ); (**c**) Down-supply up-return type window-attached-jet (DSUR-WAJ); (**d**) Down-supply up-return type vertical jet (DSUR-VJ).

**Figure 11:**
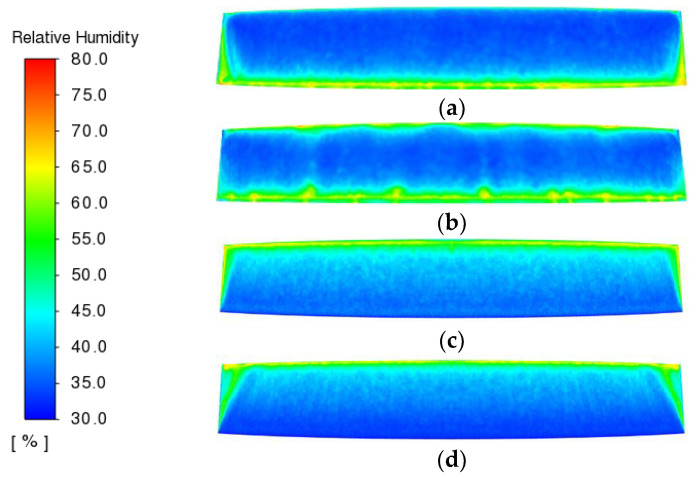
Relative humidity distribution under different air supply methods. (**a**) Top-supply down-return type window-attached-jet (TSDR-WAJ); (**b**) Top-supply down-return type vertical jet (TSDR-VJ); (**c**) Down-supply up-return type window-attached-jet (DSUR-WAJ); (**d**) Down-supply up-return type vertical jet (DSUR-VJ).

Figure 9, Figure 10 and Figure 11 show the distribution of PMV, DR and relative humidity for the different air supply methods, respectively.

As shown in Figure 9, the PMV value for the different air supply methods ranged from −0.5 to 0.3 and the driver’s comfort level was between a little warm and a little cool. Specifically, Z = 1.0 m is the shoulder area and Z = 0.2 m is the foot area of the driver. The “Top-supply down-return type window-attached-jet” air supply resulted in the lowest PMV values in the driver’s foot area, while the “Top-supply down-return type vertical jet” air supply resulted in the highest PMV values in the driver’s shoulder area. Compared with the previous two air supply methods, there was less difference in PMV at Z=1.0 m surface and Z = 0.2 m surface for the “Down-supply up-return type” air supply method, and the comfort level was higher. In contrast, at an air supply velocity of 1 m/s, an air supply temperature of 297 K and an air supply relative humidity of 30%, the “Down-supply up-return type vertical jet” air supply method created better thermal comfort for the driver.

As shown in Figure 10, the DR values for the different air supply methods ranged from 0 to 75. In particular, the “Top-supply down-return type window-attached-jet” and “Top-supply down-return type vertical jet” air supply methods result in an excessive sensation of air blowing in the driver’s foot area. The “Down-supply up-return type vertical jet” air supply method causes a greater sensation of air blowing in the shoulder and the foot area for drivers in the middle of the cockpit. The “Down-supply up-return type window-attached-jet” air supply method gives the driver less sensation of air blowing in both the shoulder and foot areas. In contrast, at an air supply velocity of 1 m/s, an air supply temperature of 297 K and an air supply relative humidity of 30%, the “Down-supply up-return type window-attached-jet” air supply method creates a better sensation of air blowing for the driver.

As shown in Figure 11, the relative humidity values of the windscreens under different air supply methods are distributed between 30% and 80%, all of which meet the design requirements for anti-fog. The “Down-supply up-return type vertical jet” has the best anti-fog effect, with only a small part of the upper side of the windscreen and the two ends of the windscreen having a slightly higher relative humidity. The “Down-supply up-return type window-attached-jet” is the second most effective.

**Figure 12:**
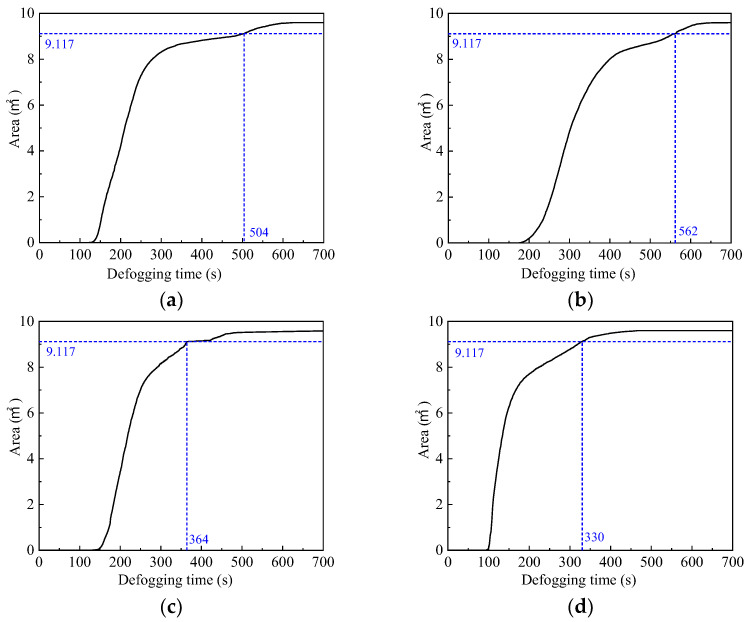
Defogging curves under different air supply methods. (**a**) Top-supply down-return type window-attached-jet (TSDR-WAJ); (**b**) Top-supply down-return type vertical jet (TSDR-VJ); (**c**) Down-supply up-return type window-attached-jet (DSUR-WAJ); (**d**) Down-supply up-return type vertical jet (DSUR-VJ).

The whole windscreen area is 19.194 m^2^, and half of the windscreen area is 9.597 m^2^. The basic requirements of defogging are considered to be met when the defogging area is greater than 9.117 m^2^. As can be seen from Figure 12, the “Down-supply up-return type vertical jet” air supply method has the best defogging effect, reaching the defogging requirement at 330 s. The “Top-supply down-return type vertical jet” air supply method is the least effective, with a defogging time of 562 s. Therefore, the “Down-supply up-return type vertical jet” air supply method is, therefore, the most effective at an air supply velocity of 1 m/s, an air supply temperature of 297 K, and an air supply relative humidity of 30%, thus providing the best guarantee of driving safety.

## 5. Optimization of Air Supply Parameters Based on Entropy Weight–TOPSIS Method

### 5.1. Entropy Weight–TOPSIS Method

TOPSIS is an acronym for technique for order preference, which means “Preference for Ordering by Similarity to an Ideal Solution” [22]. The alternative that is similar to the positive ideal solution and furthest from the negative ideal solution is considered to be preferred. The TOPSIS method involves an indicator weight. Two types of methods are commonly used to determine the indicator weights; one is the subjective determination method, such as the Delphi method and the empirical judgment method. The other is the objective determination methods, such as principal component analysis and the entropy-weighting calculation method. In this paper, the entropy-weighting method was used to determine the weights of the indicators.

The entropy-weighting method is a more accurate and objective representation of the weighting results of an indicator. The more volatile the indicator, the lower the entropy weight of the indicator, which means that the indicator has a significant impact on the evaluation objective. Therefore, more weight will be given. The entropy weight–TOPSIS method consists of the following main steps.

1. Construction of the original data matrix

The original data matrix is first created as follows.

(4) $$X=\left[\begin{array}{cccc} x_{11} & x_{12} & \cdots & x_{1m}\\ x_{21} & x_{22} & \cdots & x_{2m}\\ \vdots & \vdots & \vdots & \vdots \\ x_{n1} & x_{n2} & \cdots & x_{nm} \end{array}\right]$$

where *m* is the number of nodes in the suitability evaluation, *n* is the number of factors, and *x_ij_* is the analytical value of each sample parameter.

Then, a trend transformation of the evaluation indicators is carried out. There are three common types of evaluation metrics, namely extra-large, extra-small and intermediate. The TOPSIS method requires homogeneous trends for each indicator and requires the conversion of other types of indicators into extra-large or extra-small indicators. In practice, a uniform positive transformation is usually applied to all indicators. Extra-small and intermediate indicators are converted, while extra-large indicators remain the same.

When the parameter of the extra-small indicator is an absolute number indicator, the inverse method is used. The transformation formula is as follows.

(5) $$y_{ij}=\frac{1}{x_{ij}}$$

When the parameter of the extra-small indicator is a relative number indicator, the difference method is used. The transformation formula is as follows.

(6) $$y_{ij}=1-x_{ij}$$

If the meaning of the intermediate indicator is as close to a fixed value *α_j_* as the better, the transformation formula is as follows.

(7) $$y_{ij}=\alpha_{j}-\left|x_{ij}\right.-\left.\alpha_{j}\right|$$

If the meaning of the intermediate indicator is as far away from a fixed value *β_j_* as the better, the conversion formula is as follows.

(8) $$y_{ij}=\beta_{j}+\left|x_{ij}\right.-\left.\beta_{j}\right|$$

The matrix after the positive transformation is written as *Y*. The matrix is as follows.

(9) $$Y=\left[\begin{array}{cccc} y_{11} & y_{12} & \cdots & y_{1m}\\ y_{21} & y_{22} & \cdots & y_{2m}\\ \vdots & \vdots & \vdots & \vdots \\ y_{n1} & y_{n2} & \cdots & y_{nm} \end{array}\right]$$

2. Normalization of the initial matrix

The elements of the matrix *Y* are normalized by the Z-Score. The equation is as follows.

(10) $$z_{ij}=\frac{y_{ij}}{\sqrt{\sum_{i=1}^{n}y_{ij}{}^{2}}}$$

where *i* = 1, 2, 3, …, n; *j* = 1, 2, 3, …, m.

The normalized decision matrix can be expressed as follows:

(11) $$Z=\left[\begin{array}{cccc} z_{11} & z_{12} & \cdots & z_{1m}\\ z_{21} & z_{22} & \cdots & z_{2m}\\ \vdots & \vdots & \vdots & \vdots \\ z_{n1} & z_{n2} & \cdots & z_{nm} \end{array}\right]$$

3. Entropy-weighting determination

Entropy weighting is a method of determining the weight of an indicator from the value of the evaluation indicator under objective conditions. The entropy value of the *j*-th indicator is determined by Equations (12) and (13) as follows:

(12) $$e_{j}=-\frac{1}{\ln m}\sum_{i=1}^{m}p_{ij}\ln p_{ij}$$

(13) $$p_{ij}=z_{ij}/\sum_{i=1}^{m}z_{ij}$$

Index weight of the assessment matrix is calculated based on entropy weight, which is Equation (14) as follows:

(14) $$\omega_{j}=d_{j}/\sum_{j=1}^{n}d_{j}=\left(1-e_{j}\right)/\sum_{j=1}^{n}\left(1-e_{j}\right)$$

where $\omega_{j}$ is the weighting of each factor. $d_{j}$ is the information utility value.

4. Calculation of the Euclidean distance

The positive ideal solution and the negative ideal solution are determined first. The equation is as follows.

(15) $$z^{+}=\left(z_{1}{}^{+},z_{2}{}^{+},\cdots ,z_{m}{}^{+}\right)$$

(16) $$z^{-}=\left(z_{1}{}^{-},z_{2}{}^{-},\cdots ,z_{m}{}^{-}\right)$$

where

$$z_{j}{}^{+}=\underset{1\leq i\leq n}{\max}\left\{\left.z_{ij}\right\}\right.,z_{j}{}^{-}=\underset{1\leq i\leq n}{\min}\left\{\left.z_{ij}\right\}\right.,j=1,2,\cdots ,m$$

Then, the Euclidean distance of each alternative from the positive ideal solution and the negative ideal solution is calculated.

(17) $$d_{i}{}^{+}=\sqrt{\sum_{j=1}^{m}\omega_{j}{\left(z_{ij}-z_{j}{}^{+}\right)}^{2}},,i=1,2,\cdots ,n;j=1,2,\cdots ,m.$$

(18) $$d_{i}{}^{-}=\sqrt{\sum_{j=1}^{m}\omega_{j}{\left(z_{ij}-z_{j}{}^{-}\right)}^{2}},,i=1,2,\cdots ,n;j=1,2,\cdots ,m.$$

5. Calculation of the closeness coefficient

(19) $$c_{i}=\frac{d_{i}{}^{-}}{d_{i}{}^{-}-d_{i}{}^{+}},i=1,2,\cdots ,n.$$

The higher the value of *c_i_*, the closer the object to be evaluated is to the best point, and the better the solution.

### 5.2. Defogging and Comfort Results

The air supply studied in this paper involves four air supply parameters (air supply method, velocity, temperature, and relative humidity), and orthogonal analysis is used to investigate the above influencing factors. Specifically, the air supply methods are TSDR-WAJ, TSDR-VJ, DSUR-WAJ, and DSUR-VJ; the air supply velocity is 1 m/s, 1.5 m/s, and 2 m/s; the air supply temperature is 297 K, 299 K, and 301 K; and the air supply relative humidity is 30%, 35%, and 40%, respectively. The above parameters are orthogonalized to obtain a total of 108 combinations of air supply parameters. The comfort and anti-fog properties are calculated for all operating conditions and 108 stationary calculations can be obtained. In addition, the defogging time is calculated for all operating conditions and 108 non-stationary calculations can be obtained. The performance results are shown in Table 4. (For reasons of space, results are only given for some of the air supply methods).

### 5.3. Optimization of Air Supply Parameters

In order to accurately evaluate the combined characteristics of anti-fog, defogging, and comfort for each operating condition, the TOPSIS algorithm is used in this paper for calculation and evaluation. Firstly, the categories of the six evaluation indicators mentioned above are determined. The PMV average (Z = 1 m, Z = 0.2 m) is the intermediate type; the closer its value is to 0, the better the thermal comfort. The DR average (Z = 1 m, Z = 0.2 m) is the extra-small type; the smaller its value, the weaker the blowing sensation, the better the thermal comfort. The relative humidity is the extra-small type; the smaller its value, the lower the possibility of windscreen fogging, and the higher the driving safety. The defogging time is the extra-small type; the smaller its value, the faster the defogging efficiency, and the higher the driving safety.

In this paper, the entropy weight algorithm is adopted to evaluate the comprehensive indicators. The entropy weight results are shown in Table 5.

After determining the weights of the evaluation indicators, the TOPSIS algorithm is used to evaluate the performance of the 108 combinations of ventilation parameters. The specific results are shown in Table 6. (For reasons of space, only the top five cases’ and last five cases’ results are shown)

From Table 6, when a working condition has a smaller Euclidean distance $d_{i}^{+}$ from the positive ideal solution, and a larger Euclidean distance $d_{i}^{-}$ from the negative ideal solution, it will obtain a higher $c_{i}$. The higher the $c_{i}$, the better the working condition. After the TOPSIS algorithm search, the optimal top three working conditions are as follows. The first group and second group are the DSUR-VJ air supply method. In the first group, the air supply velocity is 1 m/s, the air supply temperature is 297 K, and the air supply relative humidity is 30%. In the second group, the air supply velocity is 1.5 m/s, the air supply temperature is 297 K, and the air supply relative humidity is 30%. The third air supply method group is DSUR-WAJ; the air supply velocity is 1 m/s, the air supply temperature is 297 K, and the air supply relative humidity is 30%. Of the above preferred working conditions, the first group has the smallest $d_{i}^{+}$ and the largest $d_{i}^{-}$; thus, it obtains a higher $c_{i}$, and presents the best combined indicators. The worst working condition is the TSDR-VJ air supply method, the air supply velocity of 2 m/s, the air supply temperature of 301 K, and the air supply relative humidity of 40%.

## 6. Conclusions

In this paper, the anti-fog characteristics of the cockpit windscreen of a polar cruise ship and the comfort characteristics of the cabin are investigated under different ventilation parameters using CFD simulation methods. On this basis, the entropy weight-TOPSIS algorithm is combined to find the optimum ventilation methods and parameters. The main conclusions are as follows.

(1) Four different types of cockpit air ventilation systems are proposed, i.e., “Top-supply down-return type window-attached-jet”, “Down-supply up-return type window-attached-jet”, “Top-supply down-return type vertical jet”, and “Down-supply up-return type vertical jet”. At an air supply velocity of 1 m/s, an air supply temperature of 297 K and an air supply relative humidity of 30%, the “Down-supply up-return type vertical jet” air supply method gives the best thermal comfort and the best anti-fog and defogging effect to the driver, while the “Down-supply up-return type window-attached-jet” air supply method gives the weakest blowing sensation to the driver.
(2) After the TOPSIS algorithm search, three preferred working conditions are obtained. The first air supply method group is “Down-supply up-return type vertical jet”; the air supply velocity is 1 m/s, the air supply temperature is 297 K, and the air supply relative humidity is 30%. The second air supply method group is “Down-supply up-return type vertical jet”; the air supply velocity is 1.5 m/s, the air supply temperature is 297 K, and the air supply relative humidity is 30%. The third air supply method group is “Down-supply up-return type window-attached-jet”; the air supply velocity is 1 m/s, the air supply temperature is 297 K, and the air supply relative humidity is 30%. Of the above preferred working conditions, the first group has the smallest $d_{i}^{+}$ and the largest $d_{i}^{-}$; thus, it obtains a higher $c_{i}$, and presents the best combined indicators.
(3) The results can be useful to provide suggestions for the future design of windshield anti-fog and defogging systems in polar cruise ship. Based on this, more different air supply methods can be considered in future research on the effect of defogging. At the same time, more influencing factors such as pollutant concentration indicators can be further considered in the evaluation system of cabin environmental control in the subsequent research process.

## Figures and Tables

**Table 1:** The specific parameters of the model.

Components	Color	Sizes
Cabin	Gray	Extension in length 14.9 m, extension in width 8.5 m, height 2.7 m
Top supply air inlet	Light green	Extension in length 10.3 m, width 0.12 m
Top supply air outlet	Dark green	(Length × width) 0.36 m × 0.36 m
Down supply air inlet	Light red	Extension in length 10.3 m, width 0.12 m
Down supply air outlet	Dark red	(Length × width) 0.36 m× 0.36 m
Windscreen	Blue	Extension in length 10.3 m, height 1.7 m
Drivers	Yellow	The height of the driver in the seated position is 1.3 m, which is simplified according to the real-life scanning model
Operating console	Purple	Extension in length 9.3 m, extension in width 2.8 m, height 0.8 m
Chairs	Black	Adapted to the driver’s size

**Table 2:** The boundary conditions.

Boundary	Boundary Type and Parameter Settings	
Models	Energy equations	On
	Turbulence model	RNG k-ε
	Species transport	On
Materials	Mixture	Air and water vapor
Inlet	Boundary type	Velocity inlet
Outlet	Boundary type	Pressure outlet
	Ambient temperature	300.15 K
Sidewalls, ceilings, floors, operating tables, chairs	Boundary type	Insulation wall
Drivers	Boundary type	Constant temperature wall
	Wall temperature	304.15 K
Windscreen	Boundary type	Wall
	Convection heat transfer coefficient	19.65 W/(m^2^·K)
	Ambient temperature	243.15 K

**Table 3:** PMV scale.

**Thermal Comfort Indicators**	Cold	Cool	A Little Cool	Moderate	A Little Warm	Warm	Hot
**PMV**	−3	−2	−1	0	1	2	3

**Table 4:** Performance assessment results.

Air Supply Parameters	Z = 1 m PMV	Z = 0.2 m PMV	Z = 1 m DR	Z = 0.2 m DR	Relative Humidity (%)	Defogging Time (s)
TSDR-WAJ (1 m/s—297 K—30%)	0.074	−0.152	4.396	26.211	46.059	504
TSDR-VJ (1 m/s—297 K—30%)	0.281	−0.033	6.688	28.283	43.820	562
DSUR-WAJ (1 m/s—297 K—30%)	0.108	0.033	1.421	9.739	39.505	364
DSUR-VJ (1 m/s—297 K—30%)	−0.043	−0.027	9.688	7.531	38.642	330

**Table 5:** Entropy weight of the factors.

Indicators	Information Entropy Value e	Information Utility Value d	Weighting Factor w
The importance of Z = 1 m PMV	0.9979	0.0021	17.51%
The importance of Z = 0.2 m PMV	0.9983	0.0017	14.45%
The importance of Z = 1 m DR	0.9992	0.0008	6.74%
The importance of Z = 0.2 m DR	0.9970	0.0030	24.74%
The importance of relative humidity	0.9977	0.0023	19.29%
The importance of defogging time	0.9979	0.0021	17.26%

**Table 6:** Evaluation results.

Ventilation Parameters	$d_{i}^{+}$	$d_{i}^{-}$	$c_{i}$	Priority
DSUR-VJ (1 m/s—297 K—30%)	0.053	0.387	0.881	1
DSUR-VJ (1.5 m/s—297 K—30%)	0.064	0.383	0.857	2
DSUR-WAJ (1 m/s—297 K—30%)	0.064	0.377	0.855	3
DSUR-VJ (1 m/s—299 K—30%)	0.063	0.368	0.854	4
DSUR-VJ (1.5 m/s—299 K—30%)	0.066	0.369	0.848	5
TSDR-VJ (1 m/s—301 K—40%)	0.281	0.231	0.452	104
TSDR-VJ (2 m/s—297 K—40%)	0.295	0.232	0.440	105
TSDR-VJ (1.5 m/s—301 K—40%)	0.265	0.194	0.422	106
TSDR-VJ (2 m/s—299 K—40%)	0.274	0.200	0.422	107
TSDR-VJ (2 m/s—301 K—40%)	0.267	0.188	0.414	108

## Data Availability

Not applicable.

## Symbols and Abbreviations

e	Information entropy value	PMV	Predicted mean vote
*d*	Information utility value	DR	Draft rate
ω	Weighting factor	TSDR	Top-supply down-return
$d_{i}^{+}$	Euclidean distance of each alternative from the positive ideal solution	DSUR	Down-supply up-return
$d_{i}^{-}$	Euclidean distance of each alternative from the negative ideal solution	WAJ	Window-attached-jet
$c_{i}^{}$	Relative proximity of each alternative from the positive and negative ideal solution	VJ	Vertical jet
